# Persuasive Technology and computational manipulation: hypernudging out of mental self-determination

**DOI:** 10.3389/frai.2023.1216340

**Published:** 2023-07-04

**Authors:** Stefano Faraoni

**Affiliations:** ^1^Law Department, University of York, York, United Kingdom; ^2^Law Department, European Legal Studies, University of Turin, Turin, Italy

**Keywords:** Artificial Intelligence, Persuasive Technology, dark patterns, hypernudge, computational manipulation, right to mental self determination

## Abstract

Artificial Intelligence, unperceived, can acquire the user's data, find connections not visible by a human being, profile the users, and aim at persuading them, resulting in Persuasive Technology (PT). During the persuasive process, PT can use manipulation, finding and using routes to affect System 1, the primordial brain of individuals, in the absence of their awareness, undermining their decision-making processes. Multiple international and European bodies recognized that AI systems could use manipulation at an unprecedented degree via second-generation dark patterns such as the hypernudge and that computational manipulation constitutes a risk for autonomy and different, overlapping, fundamental rights such as privacy, informational self-determination and freedom of thought. However, there is a lack of shared ideas regarding which fundamental rights are violated by computational manipulation and which fundamental rights can protect individuals against it. The right to be let alone and the right to hold and express a thought differ from the right to create a thought, being in control of the decision-making process and free from cognitive interferences operated by computational manipulation. Therefore, this paper argues in favor of recognizing a newly emerged fundamental right, the right to mental self-determination, tailored to the unprecedented abilities of AI-driven manipulative technologies.

## 1. Introduction

Are individuals at risk of losing their ability to self-determine their thoughts? Artificial intelligence (AI)^1^ is embedded in everyday technology, such as smartphones and social media, which have become almost essential yet also invisible and unperceived. Being as unperceived (arguably even more so) as the technology in which it is embedded, an AI can constantly acquire the user's data, find connections not visible by a human being, profile the users, and persuade them. The result is Persuasive Technology (PT).^2^ A PT not only persuades but can also manipulate by identifying and utilizing decision-making biases.^3^ This ability is considerably enhanced through the use of second-generation dark patterns and the hypernudge.^4^

Algorithmic-driven manipulative techniques are different from any form of persuasion humans can possibly exercise or be subject to (Pascal, 2018; Rose and MacGregor, 2021). They differ in the quantity of information that can be acquired on the manipulation target and in the ability to identify links in the information, links not identifiable by a human. They are different in the ability to create a cognitive profile of an individual, being able to identify not solely personal information such as gender, race, age and place of residence of an individual but also habits, visited places, relationships, preferences and cognitive processes, knowing more about the individual than the individual themselves. The knowledge acquired is used to modify and induce attitudes and behaviors, modifying the online experience according to the acquired cognitive profile of the individual. Algorithmic-driven manipulative techniques are far from the gentile nudge theorized by Sunstein (2015). A human can know that showing a picture of a baby will impact the decision-making processes of a part of the population and use this knowledge to emotionally manipulate individuals into buying products or services. An AI-driven system can know that a specific individual is pregnant and which specific emotion they feel about the pregnancy from their posts on social media, how many times showing a picture of a baby on social media brought that individual to purchase a product advertised with a picture of a baby and which other circumstances were present when the purchase was made. Moreover, an AI-driven system can know which specific cognitive biases affect the decision-making processes of that individual, such as the impact of the opinion of others on the decision-making processes of that specific individual. An AI-driven manipulative system can tirelessly and covertly acquire information on the user through interaction, adapt to their cognitive profile and change configuration until the target is reached. Entire websites can be reconfigured according to the individual's cognitive profile to affect individuals' decision-making processes in any field, from the purchase of a product to social and political choices (The Guardian, 2018; Zuboff, 2019).

Recent technological developments, such as GPT-4 (Open AI, 2023), have attracted the media's attention to possible uncontrolled and potentially dangerous uses of AI (The Guardian, 2023). Multiple bodies at regional and international levels have expressed concerns regarding PT's unprecedented ability to manipulate individuals via AI systems, with numerous suggestions that computational manipulation could undermine individual autonomy and different connected and overlapping fundamental rights, specifically the rights to privacy, self-determination, informational self-determination, freedom of thought, and the rights to hold and express an opinion.^5^ Therefore, it is recognized that individuals are at risk of losing the ability to self-determine their thoughts and actions. However, there is a lack of shared ideas regarding which fundamental rights are violated by computational manipulation and which fundamental rights can protect individuals against it.

This paper offers a review of the current concerns and legislation on computational manipulation, and argues in favor of the need to expressly recognize the right to mental self-determination, a fundamental right implied in and presupposed by different fundamental rights. The rights to withhold information and to hold and express a thought differ from the right to mental self-determination, which involves creating that thought, being in control of the decision-making processes and being free from cognitive interferences operated by emerging technology such as an AI-driven system. Manipulation has always existed (Calo, 2013). However, the manipulative abilities of an AI system are unprecedented. Therefore, an unprecedented answer is to be identified.

This paper, in Section 2, will describe what PT is and, in Sections 3, 4, will consider the concept of manipulation and how it can be used by an AI-driven system, resulting in computational manipulation that could undermine an individual's decision-making processes. Section 5 will overview the current legal concerns on computational manipulation. Finally, through the analysis conducted in Sections 6, 7, this paper argues in Section 8 the need to recognize the right to mental self-determination and extend it to include the right not to be hypernudged out of it.

## 2. What is Persuasive Technology

While attending the Conference on Human Factors in Computing Systems held in 1997 (CHI97), a behavioral psychologist, Fogg (1998), led a group of participants to study the interaction between persuasion and computers and generated the conceptual term “captology”. This term is a portmanteau, standing for “Computers As Persuasive Technology”. It refers to studies regarding the area in which computing, operating systems, and persuasion intersect.

Fogg defined PT as an “interactive computing system designed to change people's attitudes or behaviors” (Fogg et al., 1998; Fogg, 2002, p. 2) without coercion or deception. The definition has been enhanced to describe PT as a “computerized software or information system designed to reinforce, change or shape attitudes or behaviors or both without using coercion or deception” (Oinas-Kukkonen and Harjumaa, 2008, p. 202). Kampik et al. (2018) moving from this definition, considered that technology's persuasive power is perceived as dangerous in society. They and others (e.g., Berdichevsky and Neuenschwander, 1999) have raised ethical concerns regarding PT. Thus, a new definition has been conceived to embrace PT's negative aspects, considering it as “…an information system that proactively affects human behavior, in or against the interests of its users” (Kampik et al., 2018, p. 5).

By combining the previous definitions, PT can be defined as a technology that proactively influences human attitudes, behaviors, or both, changing, shaping, or reinforcing them in or against its users' interests. In other words, a PT can induce an individual to do something through reasoning or argument. A PT is a technology that can induce thoughts and a consequent choice. This kind of technology can be simple, such as a calorie counter, or very complex, such as an AI-driven system used for computational persuasion (also called compusuation; Atkinson, 2006, p. 117).

## 3. Persuasion and manipulation

It shall now be noted that persuasion can result from *peithenanke*, which, in rhetoric, consists of winning over the audience using non-transparent methods, such as manipulation (Ehninger, 1972; Fafner, 1997; Gram-Hansen, 2019).

Persuasion can be based on proper reasoning or on strategies to reach the primordial brain. According to Kahneman, drawing on the Elaboration Likelihood Model (ELM) of persuasion (Petty and Cacioppo, 1986, 2012), people have two decisional systems: one more primordial (System 1) (Petty and Cacioppo, 1986), which operates in an instinctual and subconscious way through cognitive biases, and a second (System 2), more rational, that has access to more cognitive resources (Kahneman, 2011). Using routes to affect System 1 can result in manipulation.

This article does not intend to deeply discuss the concept of manipulation, which has been the object of debate in different fields (Barnhill, 2014; Noggle, 2020; Jongepier and Klenk, 2022). This article intends to rely on some elements of manipulation that have been considered in the current debate and are relevant from a legal and fundamental rights perspective. According to Wilkinson, “manipulation is a kind of influence that bypasses or subverts the target's rational capacities” (Wilkinson, 2013; Coons and Weber, 2014, p. 11). Raz suggests that manipulation perverts how an individual makes “decisions, forms preferences or adopts goals” (Raz, 1986, p. 377). Sunstein considers manipulation an “action that does not sufficiently engage or appeal to people's capacity for reflective and deliberative choice” (Sunstein, 2015, p. 1). Susser described online manipulation as “applications of information technology that impose hidden influences on users by targeting and exploiting decision-making vulnerabilities” (Susser et al., 2018, p. 29).^6^ Despite different possible definitions of it, the kind of manipulation relevant to this analysis is characterized by two elements: it is hidden and exploits cognitive vulnerabilities in the decision-making processes of individuals (System 1).

A PT can find and use routes to affect System 1 covertly. Targeting and exploiting decision-making vulnerabilities, a PT can result in a manipulative technology, as discussed in the following section.

## 4. Computational manipulation

It is now relevant to expand on the understanding of PT's ability to manipulate and link it with AI's role in affecting and undermining the decision-making processes. The concepts of nudge, dark pattern and hypernudge will help identify the peculiarities of computational manipulation, a manipulative process driven by an AI. The mentioned concepts will be addressed in Sections 4.1, 4.2 and will be used to state the impact of computational manipulation on the decision-making processes of individuals in Section 4.3.

### 4.1. Nudges

A PT can use a *nudge*, which can be described as a choice architecture that modifies an individual's behavior predictably. The modification happens without forbidding any options or significantly changing an individual's economic incentives (Mills, 2020). An example of this architecture can be found in the cafeteria owner who positions the salad in front of a dessert to nudge individuals to eat healthy food in a public health campaign. Following a cognitive bias, individuals are more likely to choose the salad if it is more easily reachable (Sunstein, 2015; Yeung, 2017).

A nudge can be imagined as a piece of architecture, a structure, or a funnel that can be built to make, between the possible choices of the individuals targeted by the nudge, one choice more likely to happen. The “decisional choice context” can be designed to influence human decision-making toward particular directions pre-chosen by the designer (Yeung, 2017, 2.2).

According to Caraban et al. (2019), 23 ways to nudge can be identified, and the nudges can be perceived or not. Hansen and Jespersen (2013) divide nudges into four categories, which rely on two variables. The first variable is the kind of thinking engaged, which can be automatic (System 1) or reflective (System 2) (Kahneman, 2011). The second variable is the nudge's transparency, which means that the intentions behind the nudge can be perceived by the user or cannot be perceived (see also Mertens et al., 2022 on the effectiveness of nudges).

Therefore, a nudge can affect System 1 or System 2, or both, and the presence of a nudge can be perceived or unperceived. Following Susser's definition, if a nudge affects System 1 and its presence is not perceived, it can be considered manipulative (Sunstein, 2015, p. 1; Susser et al., 2018).^7^

### 4.2. Dark patterns and the hypernudge

As considered above, a nudge is a piece of architecture, a structure. To configure it, the designer must have knowledge concerning the existence of cognitive shortcuts, therefore, of people's cognitive biases. Additionally, the designer must understand how these shortcuts can be triggered to modify people's behavior in a chosen, predictable way.

Nudges can occur in the digital context as much as in the analog world. In the digital context, nudges evolved and became more powerful. According to the Eurostat data, the number of individuals who ordered or bought goods or services for private use online increased during the past 10 years (Eurostat, 2023). The increased use of online services has brought correspondent knowledge on possible peripheral routes to System 1 and the success of specific nudges regarding the users. This knowledge is acquired and used to persuade in the digital context. The same knowledge, however, can be used aiming not at persuading but manipulating the users via *dark patterns*.

Dark patterns are “practices in digital interfaces that steer, deceive, coerce, or manipulate consumers into making choices that often are not in their best interests” (EISMEA, 2022, p. 20). They are nudges based on *peithenanke* that operate online (Bösch et al., 2016; Mathur et al., 2019). According to the “Behavioral study on unfair commercial practices in the digital environment” published by the European Innovation Council and SMEs Executive Agency (EISMEA) in 2022, the design of online interfaces brings a new capability to persuasion and *peithenanke*, different from human-human interaction (EISMEA, 2022, p. 20). A dark pattern embeds knowledge about heuristics and cognitive biases in the technology. The knowledge is used to neutralize cognitive responses and defenses to attempted persuasion (Friestad and Wright, 1994). Online interaction absorbs a part of an individual's cognitive resources, leaving consumers in what has been called a *state of flow* (EISMEA, 2022, p. 22). In this state, individuals are absorbed by an experience that is genuinely satisfying, and their persuasion knowledge is neutralized (EISMEA, 2022, p. 22). Therefore, while individuals would be able to activate defensive mechanisms during a face-to-face persuasion or manipulation attempt, during the online experience, they are not.

Moreover, it shall be considered that blending technology, such as an AI-driven system and a nudge, has unprecedented consequences. A human being can have a certain amount of knowledge, which human nature limits, regarding which cognitive biases affect individuals. The cafeteria owner of the previous example knows that positioning a salad in front of a dessert will not affect all the customers, but just some of them, and in different ways. However, what would happen if the cafeteria owner had more knowledge and knew which specific cognitive biases trigger every customer entering the cafeteria and if the same owner adapted the environment accordingly in real-time? The nudge would be more effective toward every single individual.

Susser explains this concept with a representative example. In the classic theory of nudge, questions may arise as the following one: shouldn't the cafeteria food items be positioned in a way that nudges individuals to select healthy food? Susser, instead, changes the perspective. In the online world, a different question should be asked: “What if the cafeteria were arranged differently for every person who walked in the door?” (Susser, 2019, p. 404). Rearranging a cafeteria in real-time according to the preferences of every individual entering the door is impossible in the physical world. However, changing a site's configuration according to the user is commonplace in the digital world. It is possible to change the options available to the single user, the digital environment in which the user operates, and constantly change it and frame it producing different architectures for different users and even for the same user at different moments. This phenomenon is well-known as adaptive user interfaces (Browne, 2016). A human being cannot have this knowledge and capacity to adapt. Instead, an AI-driven algorithm analyzing and processing vast data sets, commonly known as Big Data, has the described ability.

Consequently, when dark patterns are applied and used by an AI, their power increases, bringing to existence second-generation dark patterns and what Yeung (2017, p. 122) defines as “hypernudge”. Yeung focuses on Big Data decision-making technologies, able to acquire a vast amount of data on single users and find links between data items not otherwise observable. By using the knowledge acquired, these technologies can channel the response and decisions of the user in directions chosen by the “choice architect” (Yeung, 2017, p. 122), according to and adapting to the users' profile. According to Yeung, the persuasive process differs when a nudge is used with Big Data-driven technology. Degli Esposti (2014) considered that the hypernudge allows nudges to operate dynamically via real-time data feeds used to personalize the outputs according to the user's actions. This mechanism is a continuous feedback loop. The output feeds the input, constantly reconfiguring the choice architecture in real-time, according to the users' actions and following their interactions.

Therefore, a second-generation dark pattern such as the hypernudge, even if based on a simple nudging process, collects its real power from the ability to determine algorithmically correlations between data not observable through human cognition. In the case of computational manipulation, an AI can create an extremely accurate cognitive profile of a single user. The profile an AI system creates and uses is not simply made of name, surname, sex, gender, residence or general preferences of an individual, but of their cognitive filters and biases and their shortcuts to System 1. Determining what could affect the decision-making processes of individuals is not based on a guess, as in a human-to-human interaction. The computational data analysis identifies what will affect individuals' decisional processes in System 1. Using its unprecedented power, the AI can continuously reconfigure the user's environment, changing and constantly evolving and matching the user's data with statistical data based on the population, configuring the possible choices of the user to influence their decisions.

According to all the above, it can be stated that the hypernudge can empower dark patterns exponentially, resulting in second-generation dark patterns. An AI can acquire and exploit the cognitive biases of a single individual, using them to change and shape the desired behavior chosen by the choice architect in an unprecedented way, consistently different from a simple nudge.

### 4.3. The impact of AI-driven PT on decision-making processes

It has to be considered now which impact this unprecedented ability might have on individuals' decision-making processes. Even if individuals cannot be assumed to make a rational or the best choice (Mik, 2016), a choice should be considered a choice only if it is possible to justify and explain it by reference to reason (Berlin, 1969; Yeung, 2017, p. 124). An hypernudge can undermine the decision-making processes in an unprecedented way, given its unprecedented abilities. An hypernudge can undermine the ability to create thoughts and make choices with unprecedented power and accuracy.

In their nature, second-generation dark patterns such as hypernudges are made of complex machine learning algorithms, enhancing their opacity and the possibility of abuse (Yeung, 2017, p. 123). The unperceived algorithmic analysis of the user constantly expands the profile in a continuous acquiring information and nudging loop, tirelessly pushing toward the result chosen by the choice architect. Second-generation dark patterns lack transparency on their existence and mechanism's functioning (Pasquale, 2006; Bracha and Pasquale, 2007). As Susser (2019, p. 403) stated, adaptive choice architectures can subtly guide individuals toward certain ends in a way that can be defined as “transparent”, by which they mean users cannot identify the existence of an hypernudge, seeing literally through it. Once individuals are used to technology, they no longer look at it as a tool to reach a purpose. Still, they look through it, seeing the information and activities behind the technology but not the technology itself. Therefore, technology becomes invisible, and a threat resides with this kind of transparency (Susser, 2019, p. 403). Moreover, the hypernudge relies on the technical possibility of a highly personalized choice environment designed to adapt to an individual's cognitive style and to create personalized routes to affect System 1. An hypernudge is far from a gentle nudge, as presented by Sunstein (2015), and it is characterized by what has been called *aggravating factors* of manipulation (Jongepier and Klenk, 2022).

According to Susser et al. (2018, p. 29)'s definition identified in Section 3, manipulation is the “application of information technology that imposes hidden influences on users by targeting and exploiting decision-making vulnerabilities”. An hypernudge matches this definition, consisting of a hidden infrastructure that can apply a hidden influence and aims to exploit decision-making vulnerabilities. Therefore, an hypernudge is powerful, unprecedented, possibly manipulative and transparent (in the meaning of non-seeable). The power of an hypernudge can interfere with the choices of individuals, which are shaped by an algorithm in decisions hypernudged for them, according to their cognitive profile. An AI-driven manipulative system can exploit individuals' cognitive biases and undermine their decision-making processes. Arguably, this can substantially negate an individual's right to self-determine their thoughts.

## 5. An overview of the existing concerns and legislation regarding computational manipulation

This section will describe the current international and European legal debate regarding computational persuasion, second-generation dark patterns, computational manipulation and the relevance of their use from a legal perspective. This section outlines how multiple international and European entities are beginning to take computational manipulation into account, recognizing its existence and raising similar concerns about its use and impact on the decision-making processes of individuals and fundamental rights.

### 5.1. Existing concerns on computational manipulation

Multiple international and European bodies have recognized the existence of second-generation dark patterns and computational manipulation. They have stated that their presence leads to the possible infringement of a right to autonomy, along with multiple other related (and overlapping) rights.

The United Nations Office of the High Commissioner for Human Rights (OHCHR) described computational manipulation as a challenge capable of putting human dignity, autonomy and privacy at risk, being able to use surveillance, analysis, prediction and manipulation to an unprecedented degree (OHCHR, 2018, p. 2). At the same time, PT's related issues were considered in a Note released by the UN Secretary-General (UNGA, 2018). In the Secretary-General's opinion, the use of AI and its manipulative ability, including personalisation, profiling and targeting, has an impact on autonomy. According to the Secretary-General, a PT can endanger human autonomy by interfering with knowledge, choice and control, supplanting, manipulating or interfering with the ability of an individual to form and hold opinions, access ideas, or express them (UNGA, 2018, p. 19). The “Resource Guide On Artificial Intelligence (AI) Strategies” released by the United Nations Department of Economic and Social Affairs (UNDESA, 2021) considers personalisation and profiling as possible instruments to trap individuals in an information bubble, causing different effects. The first effect is known as echo chambers (UNDESA, 2021, p. 7). According to the Resource Guide, AI algorithms can study individuals' interests and expose them repetitively to the same kind of content, reinforcing and shaping users' interests. Then, the Resource Guide underlined a second effect called the filter bubbles (Pariser, 2011; UNDESA, 2021, p. 7). An AI algorithm can narrow the scope of content users are exposed to. Users, therefore, will be exposed only to information and opinions that conform to and reinforce their beliefs without perception or exposure to different ideas. According to the Guide, filter bubbles and echo chambers impact fundamental rights. They may limit a person's right to obtain trustworthy information and form opinions freely, a necessary foundation for individuals to exercise freedom of expression (UNDESA, 2021, p. 7). In 2021, the United Nations Educational, Scientific and Cultural Organization (UNESCO) referred to manipulation and linked it to the abuse of cognitive biases in its Recommendation on the Ethics of Artificial Intelligence (UNESCO, 2021, art. 125).

Therefore, multiple UN-related bodies expressed the same concerns: while using AI to analyse, predict, shape and manipulate human behaviors, fundamental rights connected to human decision-making processes are in danger, such as autonomy, privacy, and the right to form, hold and express an opinion.

The Organization for Economic Co-operation and Development (OECD) proposed a definition of dark patterns that expressly mentions autonomy and decision-making:

“Dark commercial patterns are business practices employing elements of digital choice architecture, in particular in online user interfaces, that subvert or impair consumer autonomy, decision-making or choice. They often deceive, coerce or manipulate consumers and are likely to cause direct or indirect consumer detriment in various ways, though it may be difficult or impossible to measure such detriment in many instances.” (OECD, 2022, p. 16).

Therefore the OECD, like the UN-related bodies mentioned above, recognizes the existence of technologies able to manipulate users to an unprecedented degree, stating their capability of putting fundamental rights at risk and explicitly referring to the ability of dark patterns to subvert autonomy and decision-making.

In 2018 the EU released a strategy to regulate the use of AI centered on trust and the protection of fundamental rights (Commission, 2018). Since then, multiple entities in the European territory have recognized computational manipulation and its connection with the possible infringement of fundamental rights (Morozovaite, 2022).

According to the European Group on Ethics in Science and New Technologies (EGE), there are ongoing investigations to determine to which extent individuals are taken advantage of by using “advanced nudging techniques, profiling, micro-targeting, tailoring and manipulation of choice architectures” following “commercial or political purposes” (European Group on Ethics in Science New Technologies, 2018).

Concerns regarding targeted advertising and nudging via AI are then stated in the Briefing requested by the Internal Market and Consumer Protection (IMCO) committee for the European Parliament (2019). According to the IMCO, the combined capabilities of AI and Big Data can “restrict users' options, influence their opinions and manipulate them into making choices that do not serve their best interests” (European Parliament, 2019, p. 3). As already considered by the UNDESA in its Resource Guide, a further issue considered in the Briefing regards filter bubbles and echo chambers, described as able to endanger the right to form an opinion (Cadwalladr and Graham-Harrison, 2018). According to the IMCO, via the use of PT and nudging, there will increasingly be the possibility of taking advantage of irrationality or vulnerability in consumers, possibly leading to actual harm (European Parliament, 2019, p. 6).

The MSI-AUT Study for the Council of Europe (Council, 2019) underlined that manipulative practices have always existed; the emergence of AI applications has exacerbated their capacity (Council, 2019, p. 35). In the opinion of the MSI-AUT, the abilities of manipulative technologies have a relevant impact on the possibility of highly effective subtle manipulation with severe consequences for autonomy, cognitive sovereignty, and freedom of expression and information (Council, 2019, p. 8, 35). According to the MSI-AUT, persuasive digital technologies “can be used to manipulate and deceive individuals, thus interfering with both informational and decisional privacy” (Council, 2019, p. 35).

In line with the abovementioned concerns, the Committee of Ministers also emphasized that AI can predict choices. Moreover, an AI can influence emotions and thoughts, and it also possesses the ability to alter an anticipated course of action. This influence can happen subliminally (Council, 2019, n. 8). Consequently, in the Committee's opinion, PT impacts cognitive autonomy and the right to take decisions (Council, 2019, n. 9).

According to the European High-Level Expert Group on AI, PT impacts individuals' freedom to make decisions for themselves (High-Level Expert Group on Artificial Intelligence, 2019, p. 10). The Experts refer to manipulation (High-Level Expert Group on Artificial Intelligence, 2019, p. 12) as a threat to individual autonomy, self-determination, freedom of thought and privacy (High-Level Expert Group on Artificial Intelligence, 2019, p. 16).

In 2022, the European Data Protection Board (EDPB, 2022) published guidelines on dark patterns. In the opinion of the EDPB, “dark patterns aim to influence users' behavior and can hinder their ability to protect their data and make conscious choices” effectively (EDPB, 2022, p. 2).

Finally, the behavioral study released by the EISMEA for the European Commission in 2022 contains a detailed history of dark patterns and their taxonomy (EISMEA, 2022). According to the study, “dark patterns and manipulative personalisation can lead to financial harm, loss of autonomy and privacy, cognitive burdens, and mental harm” (EISMEA, 2022, p. 6).

Therefore, as considered by multiple UN-related bodies and the OECD, multiple European bodies recognized the existence of manipulative PT, stating its capability to put individual autonomy, self-determination, freedom of thought and privacy at risk.^8^

### 5.2. Existing and future EU legislation on PT

The previous paragraph considered multiple sources from different international and European bodies, which expressed similar concerns regarding using AI in a persuasive process and raised awareness toward computational manipulation and its possible impact on fundamental rights, being able to affect and undermine the decision-making processes of individuals.

In line with the abovementioned concerns but willing to pursue AI development, in February 2020, the Commission published the White Paper on AI, aiming to achieve a trustworthy use of AI to protect individuals' rights (Commission, 2020). Following the release of the White Paper, different pieces of legislation were released, namely the Digital Services Act (DSA) and the Digital Markets Act (DMA), together with the proposal for the Artificial Intelligence Act (AIA), the first attempt to legislate in the field of AI at the EU level. The mentioned legislation expressly refers to computational manipulation, recognizes the existence of dark patterns and underlines that they can subvert or impair user autonomy, decision-making, or choice, in line with what was expressed by different international and European bodies referred to in the previous paragraph.

The DSA takes directly into account dark patterns in Art. 25, stating that “providers of online platforms shall not design, organize or operate their online interfaces in a way that deceives, manipulates or otherwise materially distorts or impairs the ability of recipients of their service to make free and informed decisions”. Art. 26 imposes sharing information with the consumer regarding which criteria are used for personalisation, as stated in Recital 70. Further, the DSA obliges “very large platforms” and “very large search engines” to make a risk assessment regarding the potential manipulation of the users (Art. 34) by third parties. The DSA, in recital 67, states that nudging can distort or impair the recipients' “autonomy, decision-making, or choice”.

The DMA considers personalized advertising and consent in recital 37, stating the need for rules to ensure that consent to advertising is freely given. This Directive refers to the decision-making processes in Recital 70 and Art. 13(6), stating that gatekeepers should not use a user interface to “subvert or impair user autonomy, decision-making, or choice”.

The proposal for the AIA then establishes a list of prohibited AIs, following a risk-based approach. The list of banned practices comprises all those AI systems whose use is considered unacceptable because able to violate fundamental rights. The first banned practice in the AIA is the technology which can manipulate someone's behavior via subliminal techniques (Strahan et al., 2002; Trappey, 2005; Karremans et al., 2006; Brooks et al., 2012; Bermeitinger and Unger, 2013, p. 152) beyond their consciousness, as stated in Art. 5(1)(a). Article 5(1)(b), then, prohibits AI systems able to manipulate via exploiting the vulnerabilities of a specific group of persons due to their age or physical or mental disability. The AIA states that the General Data Protection Regulation (GDPR), the Unfair Commercial Practices Directive (UCPD) and the abovementioned DSA and DMA could cover other manipulative practices, such as those that are not subliminal. According to the EU, the abovementioned legislation guarantees that “natural persons are appropriately informed and have the free choice not to be subject to profiling or other practices that might affect their behavior” (AIA, 13; Mazzini and Scalzo, 2022, p. 24).

The European Commission, in 2021, also published a Guidance on the interpretation and application of the UCPD, which considers data-driven practices and dark patterns and expressly refers to manipulation.^9^ According to the Commission, the knowledge resulting from the newly emerged technological reality is superior, “based on aggregated data about consumer behavior and preferences” and on the possibility of adjusting in real-time, continuously testing the effects of the practices on consumers to “learn more about their behavior” (Commission Notice – Guidance on the UCPD, 2021, 4.2.7). The Commission stated that such practices might often occur without the full knowledge of the consumer and makes a distinction between highly persuasive advertising or sales techniques and commercial practices that may be manipulative. In the eye of the EU, computational manipulation can endanger the ability to process information (Commission Notice – Guidance on the UCPD, 2021, 4.2.7).

Therefore, the DSA, the DMA, the AIA and the Guidance on the UCPD all recognize the existence of manipulative AI. As already considered by the UN, the OECD and other European bodies mentioned above (Section 5.1), the EU clearly states that AI-driven manipulative technology can distort or impair an individual's autonomy, decision-making, or choice.

## 6. The theoretical foundation of the need for protection against computational manipulation; the right to autonomy

According to what was stated in the previous Section 5, the primary issue concerning using an AI for persuasion derives from the combined use of Big Data and AI's computational analysis power and ability to interact and adapt in real-time to the users' actions. Whilst both humans and AI can acquire data on individuals and covertly find and use routes to affect System 1 thought processes, and both can use open or unlawful persuasion (coercion, deception, and manipulation), the difference between (lawful or unlawful) human persuasion and computational persuasion resides in the computational capacities of an AI-driven system. A PT led by an AI system can be used for manipulation with unprecedented power, speed, personalisation, and accuracy. A PT can blur the line between persuasion and manipulation (OHCHR, 2018, p. 2) to an unparalleled degree, shaping thoughts (Williams, 2018, preface, XI. see also 23).

The documents mentioned in the previous section (consisting of studies, principles, recommendations, soft law, proposals, and legislation) link dark patterns and computational manipulation to the possible infringement of various fundamental rights. The documents identify these rights in the rights to privacy, informational self-determination, autonomy, freedom of thought, and the right to hold an opinion and express it.^10^

In this section, it will be considered that the abovementioned rights, in the field of computational manipulation, are an expression of the autonomy of the will, in the Kantian meaning expressed as “the property of the will by which it is a law to itself” (Henkin, 1974; Kant, 1997, p. 440; Guyer, 2003). As discussed further, the connection between the abovementioned rights in the field of computational manipulation is identifiable in the possible threat to the ability of individuals to be or remain in control of their thoughts if, via the use of technology, too much information is known regarding them. Moreover, the threat is enhanced if the information acquired is used with unprecedented power to affect the decision-making processes of individuals in the absence of their awareness.

The link between the power of technology, information and the infringement of fundamental rights has been considered in the past concerning one of the rights mentioned by the bodies referred to above (Secion 5). It was 1890 when, in an article written by Warren and Brandeis, serious concerns were expressed regarding possible fundamental rights violations connected to the quantity of information available regarding an individual due to newly emerged technology and the use of such information. These concerns brought to the identification of the right to privacy (Warren and Brandeis, 1890). Regarding the origin of the right to privacy, see Glancy (1979). On the right to privacy, see Prosser (1960), Thomson (1975), Regan (1990), Schwartz (1999), Mills (2008), Bennett (2010), Boehm (2011), and Bennett (2018). As discussed in the following Section 7, the use of AI for manipulation suggests today the need to identify a new fundamental right to mental self-determination tailored to account for the ability of AI to affect and undermine the decision-making processes of individuals.

In their article, Warren and Brandeis underlined issues similar to those later expressed by the bodies mentioned in the previous Section 5. The authors expressed concerns about recent inventions, new business models, instantaneous photographs and the pervasive intrusion of the newspapers into the private sphere, which could endanger, quoting Judge Cooley, the “right to be let alone” (Warren and Brandeis, 1890, p. 195).

Warren and Brandeis (1890, p. 205, 207) recognized that when information regarding an individual is made available to others, the mere fact that the information is revealed can influence and injure an individual's personality. Therefore, in their view, the right to privacy protects from one side a physical sphere, a personal bubble in which others are not allowed to enter and acquire information (such as the house or the correspondence), and from another side, the psychological integrity of an individual and the protection of their thoughts (Glancy, 1979, p. 2).

For Warren and Brandeis (1890, p. 198), “The common law secures to each individual the right of determining, ordinarily, to what extent his thoughts, sentiments, and emotions shall be communicated to others”. This sentence expresses the link between the right to privacy and the need to protect individualism (For a general discussion on individualism, see Mill, 1859; Infantino, 2014). The right to privacy is not solely the right to be let alone in a personal bubble in which other people are not allowed but also an expression of the right to decide for oneself and to form an individual personality (Glancy, 1979, p. 21–22). Therefore, the right to privacy has a psychological dimension.

The right to privacy was recognized as a human right and protected under Art. 12 of The Universal Declaration of Human Rights (UDHR). The formulation of Art. 12 is as follows:

“No one shall be subjected to arbitrary interference with his privacy, family, home or correspondence, nor to attacks upon his honor and reputation. Everyone has the right to the protection of the law against such interference or attacks.”

The right to privacy can also be found in the International Covenant on Civil and Political Rights (ICCPR), Art. 17, which formulation is almost identical to that of the UDHR (Taylor, 2020). In Europe, the right to privacy was recognized as a fundamental human right under Art. 8 of the European Convention on Human Rights. Art. 8 safeguards private and family life, home, and correspondence.

In these formulations of the right to privacy, a partition can be noticed corresponding to the original 1890 conception of this right: a physical sphere (referenced to in the home and the correspondence) and a psychological sphere (expressed in concepts as privacy, private life, family, honor and reputation). The European Court of Human Rights has interpreted Art. 8 ECHR as protecting, besides house and correspondence between others, a psychological sphere consisting of what the Court refers to as psychological dignity, the right to develop a personality, the right to self-determination, and the right to psychological integrity.^11^

Following the development of new technology, the right to privacy was extended to include the right to informational self-determination, another right the bodies considered in the previous Section 5 refer to. The German Federal Constitutional Court identified this right in December of 1983 as an expression of the right to self-determination.^12^ The right can be defined as “the authority of the individual to decide himself, based on the idea of self-determination, when and within what limits information about his private life should be communicated to others” (Rouvroy and Poullet, 2009, p. 3).

The right of informational self-determination has two sides. The first side is the right to receive information regarding possible intrusions in the private sphere. The other side is the right of an individual to decide, following the ideas of Warren and Brandeis, when and to what extent information about their private life (including their thoughts) can be communicated to others (Regarding the right to informational self-determination, see Emerson, 1971; Kolodner, 1994; Hannum, 1998; Quane, 1998; Rouvroy and Poullet, 2009; Fischer-Hübner et al., 2011; Van Alsenoy et al., 2014).

The right to informational self-determination was referred to in multiple documents at the European level concerning the use of AI. For example, in 2018, during the International Conference of Data Protection and Privacy Commissioners (ICDPPC), it was stated that while using an AI, the right to informational self-determination should be guaranteed by ensuring “that individuals are always informed appropriately when they are interacting directly with an artificial intelligence system or when they provide personal data to be processed by such systems” (Commission Nationale de l'Informatique et des Libertés, 2018, p. 4). In 2019, the Council of Europe, in the document “Unboxing Artificial Intelligence: 10 steps to protect Human Rights”, stated that: “The development, training, testing and use of AI systems that rely on the processing of personal data must fully secure a person's right to respect for private and family life under Article 8 of the European Convention on Human Rights, including the ‘right to a form of informational self-determination' in relation to their data” (Council of Europe, 2019, p. 11). Recommendation CM/Rec(2020)1 of the Committee of Ministers, regarding the right to informational self-determination, stated that “individuals should be informed in advance about the related data processing” and should be able to “control their data, including through interoperability” (Committee of Ministers, 2020, 2.1.2). The same Recommendation states that the right for individuals to “make themselves, their physical environment or their activities illegible to automation or other forms of machine reading or manipulation, including through obfuscation”, is an expression of informational self-determination (Committee of Ministers, 2020, 2.1.2).

The first concept at the root of the privacy-derived right to informational self-determination is that knowledge of meaningful information (such as interaction with an AI or data processing) is a pillar of self-determination. The second concept is that if an individual is observed or surveilled (possibly without their knowledge), or if information not to be shared is known to others, the self-determination process might be endangered. The right to choose freely might be inhibited (Rouvroy and Poullet, 2009, p. 9, referring to 1BVerfGE 65, 1 – Volkszählung Urteil des Ersten Senats vom 15. Dezember 1983 auf die mündlichenVerhandlung vom 18. und 19. Oktober 1983 - 1 BvR 209, 269, 362, 420, 440, 484/83 in den Verfahren über die Verfassungsbeschwerden).

What shall be underlined is that both the right to privacy and the privacy-derived right to informational self-determination have been identified and developed following the introduction of new technology or new uses of existing technologies. Moreover, both rights refer to preserving an inner psychological sphere.

The two rights have another common element. The concept of privacy and the privacy-derived notion of informational self-determination used in the field of AI are an expression of the autonomy of the will, in the Kantian meaning identified above. Autonomy is one of the rights referred to by the bodies considered in the previous Section 5 and consists of the capacity of individuals to legislate for themselves (European Group on Ethics in Science New Technologies, 2018, p. 9). Personal autonomy can be described as “personal self-governance, personal rule of the self by adequate understanding while remaining free from controlling interferences by others and from personal limitations that prevent choice″ (Faden and Beauchamp, 1986, p. 8; see also Christman, 2008).

Autonomy, as the privacy-derived right to informational self-determination, is, therefore, based on understanding (via receiving information such as the interaction with an AI and the data processing) and freedom from interference (having the right to exclude others from the private sphere) (On the concept of autonomy see Mill, 1859; Dworkin, 1982; Faden and Beauchamp, 1986; O'Neill, 2002). Informational self-determination has been considered an expression of Kantian autonomy, connected to the right to self-develop a personality (Rouvroy and Poullet, 2009, p. 10)^13^ via receiving information, holding thoughts and opinions, expressing them^14^ and dissenting from the opinion of others (Sunstein, 2005): the other rights mentioned by the international and European bodies referred to in Section 5. In expressing their right to informational self-determination, individuals exercise the right to autonomy and to participate in deliberative processes self-determinedly, without interference (Rouvroy and Poullet, 2009, p. 4, 8). Autonomy has been considered related to “privacy, voluntariness, self-mastery, choosing freely, the freedom to choose, choosing one's own moral position and accepting responsibility for one's choices” (Faden and Beauchamp, 1986, p. 7). In the field addressed in this paper, Williams considers PT to have implications for users' autonomy and addresses the fundamental right of freedom of thought as a dimension of autonomy (Williams, 2021, p. 13), together with awareness (Williams, 2021, p. 136) and reflection (Williams, 2021, p. 137).

According to all the above, in the field of computational manipulation, the rights mentioned by the UN, the OECD and the other bodies considered above, specifically autonomy, freedom of thought, the right to hold an opinion and to express it, self-determination, privacy, and informational self-determination, are connected and overlapping. All the abovementioned rights have been regarded as ethical principles for AI use (Jobin et al., 2019, p. 395). Moreover, in the field of computational manipulation, the mentioned rights have been considered as different expressions of the idea that, in interacting with an AI, human beings should have the freedom to decide for themselves, free from mental interferences such as coercion, “threats to mental autonomy and mental health, unjustified surveillance, deception and unfair manipulation” (High-Level Expert Group on Artificial Intelligence, 2019, p. 10).

These rights in the field of computational manipulation are related and overlapping. They have a common root in a comprehensive concept of Kantian autonomy. Autonomy is intended here as the right to create and be in control of thoughts, analyse information, form opinions, and make decisions accordingly, understanding the information available and excluding interference from the outside in a private mental sphere.

## 7. A possible right to mental self-determination as a protection against computational manipulation

In the previous Section 6, it was stated that the fundamental rights mentioned by the UN, the OECD, the EU and other bodies are connected and overlapping in the field of computational manipulation. It was also stated that the connection between the abovementioned rights in the field of compusuasion is to be identified in the possible threat to the ability of individuals to be or remain in control of their thoughts if, via the use of technology, too much information is known regarding them and used with unprecedented power for manipulation. As stated above, these rights are related to a comprehensive concept of autonomy.

In 1890, the right to privacy was identified following the concerns for the use of technology (instantaneous photography and the pervasive use of newspapers) and then extended to the right to informational self-determination following the evolution of technology. In the field of computational manipulation, the expression of Kantian autonomy leads to the theorisation of the possible existence of a new, autonomous right to be protected, the right to mental self-determination. This Section 7 will consider the new right's origin and constitutive elements. Section 8 will argue, in the conclusions of this paper, in favor of recognizing it.

The possibility of introducing a new right tailored to an AI's peculiarity was considered in 2017 by the Parliamentary Assembly of the Council of Europe (PACE), which suggested that a “right not to be measured, analyzed or coached” could be introduced in the AI field (PACE, 2017, art. 4). In a Study for the Council of Europe (released in two versions, one in 2018 and one in 2019), the MSI-AUT, having as a rapporteur Yeung,^15^ expressly stated the possible need for a new fundamental right in the AI field (Council, 2019, p. 34 and Appendix B).

The new possible right is identified as the right to cognitive sovereignty, the right to cognitive liberty or, in a formulation that reflects the abovementioned evolution of the right to privacy in the right to informational self-determination, the right to mental self-determination (Council, 2019, p. 34 and Appendix B).

Sententia (2004, 2013) conceptualized this right under the name of cognitive liberty. Sententia and Boire defined cognitive liberty as “the right of each individual to think independently and autonomously, to use the full power of his or her mind, and to engage in multiple modes of thought.”^16^ In the authors' view, the notion of cognitive liberty is considered from a positive perspective, the perspective of an individual that shall be free to alter their mind through drugs or neurotechnology able to expand the cognitive abilities of individuals. However, Sententia and Boire took into account also the necessity to avoid intrusions in the mind of individuals, stating that an individual shall be free from intrusion in their mind via the use of drugs (anti-depressant, attention drugs) and via direct electrical manipulation and interfacing technologies, preserving autonomy and what they call “brain privacy” (Sententia, 2013, p. 358).

The right was then taken into account by Bublitz, who argued that a new fundamental human right should be recognized, the right to cognitive liberty or mental self-determination. In his opinion, this right “guarantees an individual's sovereignty over their mind” (Bublitz, 2013, p. 9; Bublitz and Merkel, 2014: see also Ienca and Andorno, 2017; Weissenbacher, 2018; Sommaggio and Mazzocca, 2020). Bublitz, similarly to Sententia, took into account this right mainly from the perspective of neuroenhancements, the pharmaceutical improvement of the mind (Bublitz refers to Galert et al., 2009). He considered that this right should be the central legal principle and guide the regulation of neurotechnologies. In Bublitz's opinion, the right to alter an individual's mental state with neuro tools should be recognized, as well as the right to refuse to modify it (Bublitz, 2013, p. 2).

Bublitz (2013, p. 12), identifying the origin and the theoretical foundation of this right, referred to Kant (as done by this analysis in Section 6) and Mills and argued that the law presumes that individuals have free will. According to the author, if individuals are treated by criminal and contract law “as self-determined over their actions and antecedent mental states”, and if the law “holds them accountable for consequences of mind-states (…) as if they had free will”, then, the law shall grant individuals the powers derived from self-determination (Bublitz, 2013, p. 12). Accordingly, Bublitz described cognitive liberty as the right to free will. This right is implied in any legal order that considers self-determination and responsibility (Bublitz, 2013, p. 12).

As previously done by Sententia, Bublitz connected the right to cognitive liberty to the concept of privacy, to the right to develop a personality and to the right to mental integrity or mental health (Bublitz, 2013, p. 14), stating that “some rights are closely related to the idea of cognitive liberty” (Bublitz, 2013, p. 17). However, these rights cannot protect the particularities of interferences with the mind (Bublitz, 2013, p. 17). He believes “legal theory has yet to develop more fine-grained doctrines dealing with the mind and mental states” (Bublitz, 2013, p. 17). Therefore, Bublitz identified some aspects of the right to cognitive liberty, or mental self-determination. The first aspect is the liberty to change one's mind. The second aspect of the right is to shield individuals from intrusions into their minds and preserve their mental integrity (Bublitz, 2013, p. 19).

This right has been taken into account by McCarthy-Jones (2019), who considered it also from the perspective that thoughts shall not be manipulated. McCarthy-Jones underlined how the US courts have already considered the principle that thoughts should not be manipulated.^17^ The author also considered that the mentioned principle could be linked to the right to mental integrity as identified by the 2009 Charter of Fundamental Rights of the European Union, Art. 3.1 and is a principle supporting some European Court of Human Rights decisions.^18^ McCarthy-Jones (2019, p. 11) argued that the right to mental self-determination secures mental autonomy, which shall be protected by prohibiting the manipulation of thoughts.

Douglas underlined that given that many states recognize “a right against significant, non-consensual interference with one's body” (Douglas and Forsberg, 2021, p. 179), an equal right to mental integrity should be recognized against interferences “with the mind” (Douglas and Forsberg, 2021, p. 182). Douglas referred explicitly to the concept of nudge, considered a mental interference against which protection should be received (Douglas and Forsberg, 2021, p. 194).

The terms cognitive liberty, cognitive sovereignty and mental self-determination are then considered by emerging literature on the thoughts of Bublitz (Michalowski, 2020). The existing literature in this field has discussed the possible existence of neuro rights, identifiable as new human rights to protect mental processes (Yuste et al., 2017). Ienca and Andorno (2017) proposed distinguishing different aspects of the interference in the human mind and suggested considering the aspects under different rights: mental integrity, mental privacy, psychological continuity and cognitive liberty. Lavazza (2018), instead, discusses the possibility of a single right to mental integrity. The neuro rights have also been considered with a critical approach like that of Herz, which analyzed the work of the authors mentioned above and evaluated the neuro rights under existing human rights. However, Hertz (2023) argued that existing human rights should be reinterpreted to make them adhere to the reality of AI.

Following the thoughts described above, as stated at the beginning of this section, the MSI-AUT, in a Study for the Council of Europe, recognized the possible need for a new human right tailored to the unprecedented AI's abilities to interfere with the mind. According to the MSI-AUT, the new right can provide a more robust approach to protect individuals against computational manipulation. The MSI-AUT followed the thoughts of Bublitz (2020) and considered that the new right could guarantee individuals' sovereignty over their minds. In the words of the MSI-AUT, there might be the need for “a free-standing right to cognitive sovereignty (akin to the rights of data protection) which overlaps with other human rights, including those arising under Articles 8, 9, and 10” (Council, 2018, p. 79). This need will be discussed in the following and final section.

## 8. Conclusions: the need to recognize the right not to be hypernudged out of mental self-determination

Transparent in the meaning identified by Susser (Susser, 2019, p. 403),^19^ an AI can constantly acquire the user's data, find connections not visible by a human being, profile the users and their cognitive biases, and aim at persuading them.

As considered in Section 4, an AI-driven PT can use manipulation, covertly influencing individuals by targeting and exploiting their decision-making vulnerabilities. PT's ability to undermine the decision-making processes of individuals is considerably enhanced using compusuasion, the ability to persuade through AI, second-generation dark patterns and the hypernudge. Algorithmic-driven manipulative techniques are different from any form of manipulation individuals experienced in the past during an in-person persuasive process.

As considered in Section 5, multiple international and European bodies expressed concerns about PT's unprecedented ability to manipulate individuals via AI systems. Following the mentioned concerns, the EU took even the first steps to legislate against computational manipulation. At an international and European level, it is recognized that an AI system can hypernudge individuals' decision-making processes, affecting their ability to create a thought self-determinedly.

As considered in Section 6, different fundamental rights have been deemed infringed by PT's manipulative abilities, namely autonomy, freedom of thought, the right to hold an opinion and to express it, self-determination, privacy, and informational self-determination. However, there is a lack of shared ideas at the international and European levels regarding which fundamental rights are violated by computational manipulation and which fundamental rights can protect individuals against it.

One common thread is noticeable in the approach of different international and European bodies regarding computational manipulation, consisting of recognizing the possible threat to the ability of individuals to be or remain in control of their thoughts if, via the use of technology, too much information is known regarding them and used with unprecedented power for manipulation. According to the mentioned bodies, the unparalleled abilities of a second-generation dark pattern consist of analyzing Big Data, profiling, interacting, finding routes to affect System 1, adapting according to the users' cognitive profile and manipulating them into a choice hypernudged for them.

In 1890, the right to privacy was identified following the concerns for the use of new technology (such as instantaneous photography and the pervasive use of newspapers) and extended to the right to informational self-determination following the evolution of technology. As stated in Section 7, in the field of computational manipulation, the expression of Kantian autonomy leads today, in the age of AI, to identify a new, autonomous right to be protected, the right to mental self-determination. This right has its roots in an older category of fundamental rights, such as the right to autonomy. It is tailored to the unique abilities of the unprecedented emerging technology to interfere with the decision-making processes of individuals and, therefore, with how thoughts are formed, not with how they are expressed. This right should be recognized and extended to protect individuals from being hypernudged out of their mental self-determination.

Agreeing with the line of reasoning expressed by Sententia, Bublitz and others mainly from the perspective of neuroenhancements and recognized by the MSI-AUT, the right to hold a thought and express it or share it with others should be distinguished from the right to mental self-determination. This right is different because it involves creating a thought, being in control of the decision-making processes and free from cognitive interferences operated by newly emerged technology such as an AI-driven system. The right to express a thought is deprived of any validity if that thought is not self-determined. Moreover, once expressed, it shall be considered that a thought might bring consequences from a legal perspective. It can result, for example, in the expression of consent in a contract. As recognized by Bublitz, if the law regards individuals as self-determined in their actions, and considers them accountable for consequences of their state of mind as if they had free will, then the law shall recognize the right to self-determine the thoughts that are held and expressed. Agreeing with Douglas, a fundamental right should be recognized against interferences with individuals' minds, such as those perpetrated by an AI-driven PT. Further protection can then be granted at a regional and national level, with specific interventions in fields such as contract law, tort law and criminal law to allow specific remedies to individuals to be identified in possible future contributions to this field.

Therefore, in the age of AI, the fundamental right that shall be protected is a right that is implied in and presupposed by other fundamental rights, and that should be expressly recognized, the right to mental self-determination. This new right should include the right not to be hypernudged out of mental self-determination.

## Author contributions

The author confirms being the sole contributor of this work and has approved it for publication.

## Footnotes

^1^This article will not provide a definition of Artificial Intelligence which is not necessary for the purposes of the article. For a possible definition of Artificial Intelligence, see High-Level Expert Group on Artificial Intelligence “A definition of AI: Main capabilities and scientific disciplines” (December 18, 2018), 7. AI includes several techniques, such as machine learning, machine reasoning, and robotics.

^2^See Section 2.

^3^See Section 3.

^4^See Section 4.2.

^5^See Section 5.

^6^This definition will be used during the following analysis. It has also been suggested that manipulation might not always be hidden (Klenk, 2022). However, for what is relevant to this analysis, even the authors of this vision recognize that it can be hidden.

^7^See Section 3.

^8^There shall be noted that also the UK, not anymore part of the EU, released on 29/03/2023 a white paper called “A pro-innovation approach to AI regulation”, which acknowledges, in the references in points 4 and 22, the risks of computational manipulation for autonomy and decision making processes of individuals.

^9^Commission Notice – Guidance on the interpretation and application of Directive 2005/29/EC of the European Parliament and of the Council concerning unfair business-to-consumer commercial practices in the internal market (2021) OJ C 526.

^10^Please, refer to Section 5 for references and citations.

^11^ECHR, ‘Guide on Article 8 of the European Convention on Human Rights' (April 30, 2022), 28, considering *Beizaras and Levickas v. Lithuania*, § 117 on psychological dignity, *Von Hannover v. Germany* (no. 2) [GC], § 95 on the right to develop a personality, *Pretty v. the United Kingdom*, § 61 on the right to self-determination, *Vavřička and Others v. the Czech Republic [GC]*, § 261 on psychological integrity.

^12^1BVerfGE 65, 1 – Volkszählung Urteil des Ersten Senats vom 15. Dezember 1983 auf die mündliche Verhandlung vom 18. und 19. Oktober 1983 - 1 BvR 209, 269, 362, 420, 440, 484/83 in den Verfahrenüber die Verfassungsbeschwerden.

^13^The right to develop a personality is protected under the UDHR, art. 22.

^14^Protected under art. 19 of the UDHR.

^15^Karen Yeung theorized the Hypernudge. Please refer to Section 4.2 and to Yeung (2017).

^16^This definition can be found on the website of the Center for Cognitive Liberty founded by the two authors: https://web.archive.org/web/20120206215115/http://www.cognitiveliberty.org/faqs/faq_general.htm.

^17^The author cites at 10 Rennie v. Klein (1981). 653 F.2d 836 (3d Cir.)., Stanley v. Georgia (1969). 394 U.S. 557, 89 S. Ct. 1243, 22 L. Ed. 2d 542. and Ashcroft v. free speech coalition (2002). 535 U.S. 234, 122 S. Ct. 1389, 152 L. Ed. 2d 403.

^18^The author cites, at 10, Larissis and Others v. Greece (1998). 65 Eur. Ct. H.R. (ser. A) 363, 27 E.H.R.R. 329, regarding high-ranking officers who attempted to convert lower-rank soldiers to Jehova'sWitnesses and Kokkinakis v. Greece (1994). 17 E.H.R.R. 397, which refers to the so called brainwashing in a religious context.

^19^See Section 4.3.
